# Essentials of Physics

**Published:** 1894-05

**Authors:** 


ESSENTIALS OF PHYSICS.—Arranged in the form of Questions and Answers. Prepared especially for Students of Medicine. By Fred J. Brockway, M.D., Assistant Demonstrator of Anatomy at the College of Physicians and Surgeons, New York. Second Edition. Revised, with 155 Illustrations. Philadelphia. W. B. Saunders, 1894. [Price $1.00.]

As one of Saunders' Question Compends, this book needs little introduction to the medical student. The author claims nothing original, except the arrangement of a book which is a mean between some elementary books which contain too little, and the text-books which contain too much. It contains all that is essential in a course of medicine. V. H. T.

